# Mixing of Two Microbial Consortia in the Search for Stimulating Chromium Depletion

**DOI:** 10.1155/ijm/8555038

**Published:** 2025-03-31

**Authors:** Elcia Margareth Souza Brito, Paola Abigail Martínez-Aldape, Sara Lucia Toscano-Alaniz, César Augusto Caretta, Mónica Jacquelina Martínez-Ramírez, Ramón Eugenio Martínez-Ramírez, Mario Enrique Sandoval-Vergara, Sandra Ixmucamé Concha-Guerrero, Marisol Goñi-Urriza, Alma Hortensia Serafín-Muñoz, Claudia Adriana Ramírez-Valdespino, Rémy Guyoneaud

**Affiliations:** ^1^Departamento Ingeniería Civil y Ambiental (DI-CGT), Universidad de Guanajuato, Guanajuato, Mexico; ^2^Université de Pau et des Pays de l'Adour, Environmental Microbiology Group, IPREM UMR CNRS 5254, IBEAS, Pau, France; ^3^Departamento de Astronomía (DCNE-CGT), Universidad de Guanajuato, Guanajuato, Mexico; ^4^Departamento de Ingeniería, Universidad Iberoamericana, León, Guanajuato, Mexico; ^5^Centro de Investigación en Materiales Avanzados SC, Chihuahua, Mexico

**Keywords:** bacterial isolation, bacterial synergism, biofilm formation, Cr(VI) depletion

## Abstract

Two bacterial consortia (C55 and C33), obtained from an industrial residue contaminated with hexavalent chromium (Cr(VI)), were used to study the behavior of their mixture for depleting this ion in liquid media. In the absence of Cr(VI), C55 showed a greater growth rate than C33, while the latter exhibited biofilm formation. In the presence of this ion, C55 showed resistance up to 800 mg·L^−1^ and an ability to diminish up to 400 mg·L^−1^ of the Cr(VI) from the medium, while for C33, these concentrations were 400 and 200 mg·L^−1^, respectively. Bacterial synergism between these consortia was evaluated using different compound ratios (C55:C33 ratios of 1:1, 1:2, and 2:1), growing at 50, 100, and 200 mg·L^−1^ Cr(VI). The best half-lives of Cr(VI) decrease were 16, 31, and 98 h, respectively, for the 1:1 mixture. The ability of C33 and the mixed consortia to form biofilms was verified. MiSeq sequencing revealed 4 major populations for C55 (in a total of 14) and 3 for C33 (8), most of which were common. After an isolation process, 2 bacterial strains were obtained from C55 and 4 from C33. Three of these strains (QRePLB33E, similar to *Oceanobacillus profundus*; QRePLB33G, to *Shouchella clausii*; and QRePLB55C, to *Cellulosimicrobium funkei*) showed resistance to Cr(VI) and the ability to remove 100% of it at least up to 300 mg·L^−1^. Thus, synergism between different bacterial consortia obtained from the same site is possible and can improve, by complementing their capacities, both the growth rate and the ability to diminish the xenobiotic from the medium.

## 1. Introduction

Chromium is a metal that is in considerable demand by several industries, such as metallurgy, cast iron, automotive, refractory, and chemical (leather, electroplating, and wood preservation). Discharges from these industries usually contain large amounts of chromium salts, which, if not properly treated, can pose a major threat to the environment and human health. Although chromium can exist in nature in nine valence states (i.e., from −2 to +6), trivalent and hexavalent ions (Cr(III) and Cr(VI), respectively) are the majority forms, with Cr(VI) being approximately 100 times more toxic than Cr(III) [1]. This occurs mainly because Cr(VI) is highly water soluble, permeable to cell membranes, and recognized as a carcinogen and mutagen [1], which reinforces why chromium discharge into the environment should be controlled. Chromium-contaminated effluents can be treated by chemical reduction, ion exchange, precipitation, membrane technologies, etc., which are expensive and inefficient and usually produce secondary pollutants. In the search for eco-friendly alternatives, there are biotechnologies in which microorganisms are used to diminish Cr(VI) from their environment via cellular exudates and/or intracellular enzymatic reactions [2]. Some of these studies explored the potential of microorganisms using isolates [3, 4], bacterial consortia [5], or bacterial biofilms [6, 7] to decrease the amount of Cr(VI).

In nature, bacterial communities that flourish in networks are very common, and continuous interactions between microbial populations allow for great adaptation to their environment. This cooperation can implicate absolute or partial mutual dependence [8]. Microbial metabolic cooperation (through the combination of metabolic activities) that benefits both partners, called “obligate mutualistic metabolism” [9], is the typical synergistic effect observed in bacterial biofilms [10, 11]. In these biofilms, the microorganisms are embedded in a self-secreted protective matrix surrounded by immobilizing microbial cells [12]. This matrix, known as EPS (from exopolysaccharides), is composed of highly hydrated polysaccharides, proteins, lipids, and nucleic acids but also contains water-insoluble compounds such as cellulose or amyloids. Taking part in a biofilm has many advantages, such as water retention and the formation of localized chemical gradients (pH, dissolved oxygen, redox potential, concentration of nutrients and metabolic products) and microenvironments [12]. Additionally, this structure has other important characteristics, such as the retention of extracellular enzymes near cells, the capture and retention of cellular products and debris that act as reservoirs of biological compounds for microorganisms, the facilitation of synergistic effects between different species, the exchange of genes, and increased tolerance against chemicals or antimicrobial agents. All these facilities allow the occupation of new habitats and the continuous regeneration of the biofilm in response to stressful environmental conditions.

Biofilm resistance to metals has encouraged researchers to use bacterial biofilms as part of bioprocesses for Cr(VI) mitigation, typically investigating the adsorption capacity of these biofilms, the immobilization of Cr(VI), and its reduction to Cr(III) [13, 14]. On the other hand, native microorganisms, which are better adapted to their surrounding conditions, may exhibit additional adaptations that make them more efficient against xenobiotics.

Based on these assumptions, we obtained several bacterial consortia from a chromium salt extraction mine waste to apply them in a possible bioprocess for Cr(VI) treatment [15]. Here, we explore the cooperation of two consortia, C55 and C33, which were selected because of both their greater resistance and ability to decrease the amount of chromate ions from culture media and their capacity to produce EPS. Our hypothesis is that the combination of these consortia, by combining the efforts of the two communities, that is, their individual capabilities, could stimulate biofilm growth through a synergistic effect and thus improve Cr(VI) diminution.

To better understand these consortia, additional tests were performed: (a) comparison of the bacterial composition of individual consortia (C55 and C33) through metagenomic bacterial diversity analysis; (b) bacterial isolation and identification of the obtained strains; (c) analyses of the chromate ion diminution and depletion by the isolates, original and mixed consortia; and (d) biofilm formation by scanning electron microscopy (SEM). These results might provide an improved understanding of the interactions between bacterial consortia during biofilm formation in the presence of Cr(VI).

## 2. Materials and Methods

### 2.1. Prospection of the Original Microbial Consortia

**Figure 1:**
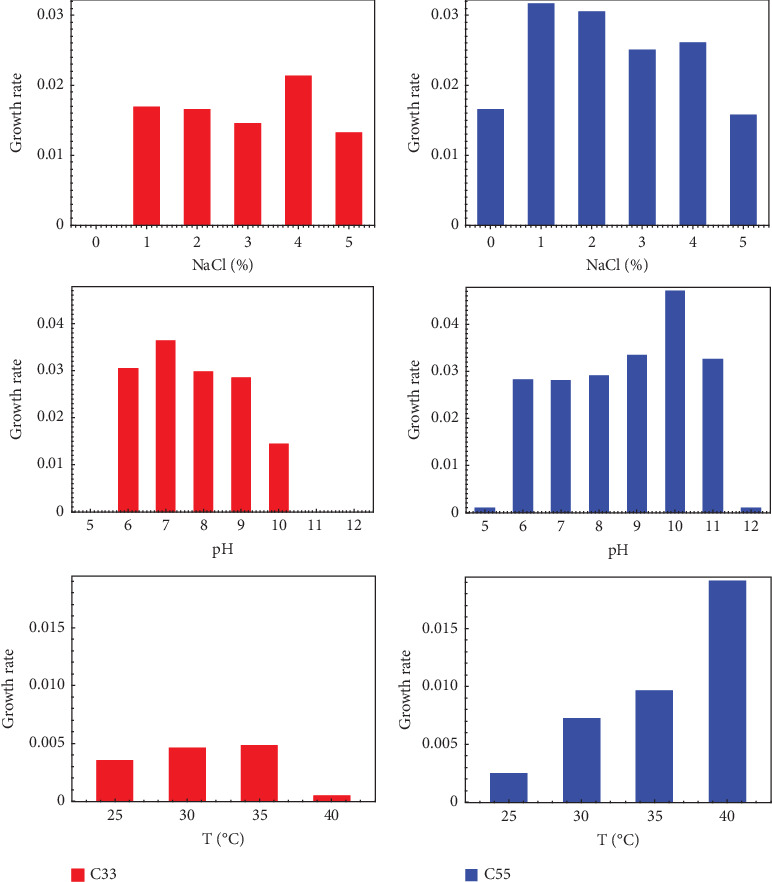
Determination of the optimal conditions for the growth of C33 and C55 original consortia: % NaCl, pH, and temperature (*T*). Experiments for C33 are in the first column (red histograms) and for C55 in the second one (blue histograms).

The original C33 and C55 consortia were recovered from chromite ore tailings located in Guanajuato State, Mexico (21°02⁣′32⁣^″^N, 101°47⁣′29⁣^″^W). Sediments collected from leach channels were used to prepare a sample suspension containing 1 g of sediments and 9 mL of liquid media. Consortium C33 was originally cultured in LB medium, while C55 was cultured in R2A medium. The LB medium contained (grams per liter) tryptone (10), yeast extract (5), and NaCl (10), while the R2A medium [16] contained (grams per liter) yeast extract (0.50), protease peptone (0.50), casamino acids (0.50), glucose (0.50), starch (0.50), Na-pyruvate (0.30), K_2_HPO_4_ (0.30), and MgSO_4_·7H_2_O (0.05). Both media were adjusted to pH 7.4 (measurements were done with a Hanna model HI98103 pH meter). Solid media were prepared by adding 15% of bacteriological agar. A serial dilution (up to 10^5^) was initially performed in liquid medium. Subsequently, 50 *μ*L of each dilution was spread onto agar plates and incubated at 35°C. Individual colonies were recovered, subcultured on a new solid medium, and later cultured in liquid media (LB, M9, and R2A). The colonies were then grown in LB liquid medium containing 50 mg·L^−1^ Cr(VI). Cell morphology was assessed using optical microscopy (OM, Zeiss Primostar) and SEM. Optimized growth conditions for both consortia were determined by Sandoval-Vergara [17] and are presented in Figure 1. Both C33 and C55 consortia are haloalkaliphilic, exhibiting optimal growth under saline, moderately basic and mesothermic conditions. The optimal conditions for C33 were identified as 4% NaCl (moderately halophilic), pH 7 (neutral), and a temperature of approximately 35°C. In contrast, C55 exhibited optimal growth at 1% NaCl (halotolerant), pH 10 (alkalophilic), and a temperature of 40°C or higher.

### 2.2. Microbial Composition of the Original Consortia

Bacterial populations inhabiting the consortia were assessed by massive sequencing of 16S rDNA. Total DNA was first extracted using a DNeasy Power Soil Pro Kit (QIAGEN) following the protocol provided by the manufacturer. Then, the V4–V5 region of the 16S rRNA gene was obtained by PCR using AmpliTaq Gold 360 Master Mix with primers 515F (0.6 *μ*M, GTGYCAGCMGCCGCGGTA) and 928R (0.6 *μ*M, ACTYAAAKGAAT TGRCGGGG), together with 1 *μ*L of DNA. DNA quality was verified by UV/Vis spectroscopy (NanoDrop ND-1000, Peq-lab, Erlangen, Germany), and the DNA sample was sent to the TANDEM Laboratory of the INRA (Institute National de la Recherche Agronomique, INP Toulouse, France). The V4–V5 region of 16S rRNA was sequenced with Illumina MiSeq technology with paired-end reads. The primers used were PCR1F_460 (position *Escherichia coli* 344–357; 5⁣′-CTTTCCCTACACGACGCTCTTCCGATCTACGGRAGGCAGCAG-3⁣′) and PCR1R_460 (position *E. coli* 784–802; 5⁣′-GGAGTTCAGACGTGTGCTCTTCCGATCTTACCAGGGTATCTAATCCT-3⁣′) [18]. Reads of 376 bp were recovered using the Galaxy FROGS pipeline bioinformatic process. The raw sequences of the present project were deposited in the Sequence Read Archive (SRA) database of the National Center for Biotechnology Information (NCBI) and assigned accession numbers associated with the project PRJNA1121098.

### 2.3. Bacterial Isolation and Identification

Firstly, the C33 and C55 consortia were cultured in modified LB liquid medium under previously determined optimal growth conditions. After three successive cultures, to ensure metabolically active bacterial populations, a serial dilution was performed (up to 10^–6^), and the 10^–6^ dilution was plated on LB solid medium using the streak plate method. The selected colonies were then subcultured successively on LB solid plates until colonies with uniform morphology were obtained. The morphology of the bacterial cells was confirmed by microscopy observations. To identify the isolates and verify the purity of axenic culture, their DNA was extracted with a Wizard Genomic DNA Purification Kit (Promega) according to the manufacturer's instructions. The amplification of the bacterial 16S rRNA gene was performed using the primers 8F and 1489R (5⁣′-AGAGTTTGATCCTGGCTCAG-3⁣′ and 5⁣′-TACCTTGTTACGACTTCA-3⁣′, respectively) [19]. For details of the PCR protocols, see Brito et al. [20]. The PCR amplicons from the isolates were sent to GATC (Germany) for Sanger sequencing. The sequences were compared to those in the NCBI database using the BLAST method. The 16S rRNA sequences of the isolates and their respective closest references were aligned with the MAFFT (multiple alignment using fast Fourier transforming) [21] program. The phylogenetic tree was constructed with MEGA 11 [22] software using the maximum likelihood method. The confidence of the phylogenetic tree was assessed by bootstrapping using 1000 resamples. The sequences obtained in this study were submitted to the GenBank database (assigned Accession Numbers PP747663–PP747668).

### 2.4. Conditions for Mixing the Original Consortia

Before selecting the culture medium for the Cr(VI) reduction experiments, all reagents from the LB and M9 media were individually tested as potential electron donors for the chemical transformation of Cr(VI) to Cr(III). Using a K_2_Cr_2_O_7_ solution at 270 mg·L^−1^ of Cr(VI), it was observed that yeast extract, protease peptone, starch, sodium pyruvate, casamino acids, and glucose reduced Cr(VI) by 2%, 22%, 25%, 49%, 74%, and 95%, respectively. Given that peptone and yeast extract caused the lowest chemical reduction of Cr(VI) to Cr(III), these reagents were selected to compose the culture medium (10 g·L^−1^ peptone and 5 g·L^−1^ yeast extract), after used in the experiments to evaluate Cr(VI) reduction by the bacterial consortia. Additionally, chemical control assays without microorganisms were also performed to confirm the observed Cr(VI) reduction was due to biological activity.

To determine the growth conditions for the mixture of consortia C33 and C55, their respective pH, NaCl concentration, and temperature ranges were considered [17], as well as the environmental conditions of the leachates where these consortia would be applied [23]. Specifically, this study is part of a bioprocess aiming to diminish Cr(VI) concentrations in leachates from chromite industrial processing, which are highly halo-alkaline (pH above 9 and salinity around 100 PSU) [23], and taking into account the information in Figure 1, a pH of 9 was selected as a compromise suitable for both consortia. For salinity, a concentration of 4% NaCl was chosen, as higher values could hinder the growth of both populations. Similarly, a temperature of 37°C was selected to avoid negative effects on the growth of the C33 consortium, as higher temperatures could reduce its viability and increase the overall cost of the bioprocess under investigation, whose results will be published elsewhere in due course. Once the culture of both C33 and C55 consortia reached the exponential phase, after approximately 48 h of incubation at 37°C, the cells were precipitated (8000 rpm for 5 min) and counted with a Neubauer chamber. Different quantities of each consortium were mixed (in proportions for C55:C33 of 1:0, 0:1, 1:1, 2:1, and 1:2—while 1:0 and 0:1 will continue to be referred to as C55 and C33; the other mixtures will be referred, in some parts of the text, as consortia C11, C21, and C12 for simplicity), maintaining a final concentration of microorganisms of 10^7^ cel·mL^−1^. Both the (a) bacterial growth of each mixture and (b) the ability to decrease Cr(VI) in the media were tested. The bacterial growth under each condition was tested on a LB medium without Cr(VI) by the turbidimetric method [24]. Taking the bacterial population growth in its exponential phase (that is, when there is a linear relation between the natural logarithm of the OD and time), we can calculate its respective growth rate (*μ*), from which we obtain the generation time (*t*_*g*_, the time necessary for the population to double its size):

(1) $$\begin{array}{c} t_{\text{g}}=\frac{\ln2}{\mu }=\frac{0.693}{\mu }. \end{array}$$

Using the same conditions described above, three Cr(VI) concentrations were tested: 50, 100, and 200 mg·L^−1^, together with a control without Cr(VI). Immediately after inoculation (10^7^ cel·mL^−1^), Cr(VI) was added using a sterile stock solution of K_2_Cr_2_O_7_ (2 g·L^−1^). Subsamples were then taken from all the experiment units for Cr(VI) determination at time zero. These systems were incubated at 37°C. Aliquots of cultures were periodically taken for Cr(VI) determination until Cr(VI) became undetectable in the supernatant. All the assays were performed in triplicate to assess reproducibility.

### 2.5. Assessment of Cr(VI) Diminution

Cr(VI) was quantified by a colorimetric method employing 5 mg·L^−1^ S-diphenyl carbazide (DPC) prepared in acetone [25]. Briefly, first 3.95 mL of distilled water was acidified with 1 mL of 1 M H_2_SO_4_, after which 100 *μ*L of the sample was added, followed by 50 *μ*L of DPC. After 10 min under obscurity, the absorbance was measured at *λ*540 nm, and the Cr(VI) concentration was quantified using a standard curve. All samples were run in triplicate. To obtain the standard curve, K_2_Cr_2_O_7_ was dried in an oven at 45°C for 24 h. A stock solution of 2000 mg·L^−1^ Cr(VI) was prepared, from which working solutions ranging from 1 to 100 mg·L^−1^ Cr(VI) (1, 2.5, 5, 10, 50, 80, and 100 mg·L^−1^, all in triplicate) were generated. The Cr(VI) concentration of these solutions was determined using the described procedure, and the corresponding absorbance at 540 nm was measured. A linear correlation was fitted between Cr(VI) concentration and absorbance, resulting in the equation *y* = 0.0052 *x* + 0.0113 with *R*^2^ = 0.9994. The minimum and maximum detection limits were established as 0.25 and 80 mg·L^−1^ Cr(VI), respectively, corresponding to absorbance values of 0.025 ± 0.006 and 0.428 ± 0.010. The quantification limit was determined to be 0.006 nm. This technique is suggested in the Mexican regulations for Cr(VI) determination in wastewater [26].

Cr(VI) diminution was assumed to be a pseudo–first-order reaction [27, 28]; then, the kinetic reaction of chromate reduction by bacterial consortia was calculated by using

(2) $$\begin{array}{c} \frac{C_{t}}{C_{0}}=e^{-kt} \end{array}$$

where *C*_*t*_ is the Cr(VI) concentration at time *t*, *C*_0_ is the initial concentration, and *k* is the constant *decay* rate calculated as the slope of the linear fit obtained by plotting ln(*C*_*t*_/*C*_0_) against time (*t*). Similar to *t*_g_, the half-life (*t*_½_) of Cr(VI) was calculated by

(3) $$\begin{array}{c} t_{½}=\frac{\ln2}{k}=\frac{0.693}{k}. \end{array}$$

### 2.6. SEM Observations

SEM observations followed the methodology described in Concha-Guerrero et al. [29] and Maldonado et al. [30]. First, fresh cultures were centrifuged (3000 rpm for 5 min), washed two times with Millonig buffer phosphate (0.1 M, pH 7.3) and then fixed in 3% glutaraldehyde Millonig buffer phosphate (0.1 M, pH 7.3) for 4 h. After the samples were washed four times in the same buffer, they were filtered through a polycarbonate membrane (0.22 *μ*m, Millipore). They were then dehydrated by increasing successively the ethanol concentration (30%, 50%, 70%, 90%, and 100%) and dried by critical point. The samples were mounted on metal stubs and coated with gold (Denton Vacuum Desk II). They were, then, sent to a specialized laboratory for carrying this analysis (Center for Research in Advanced Materials, Chihuahua, Mexico) where a JEOL JSM Model 7401F scanning electron microscope was used to generate the images.

## 3. Results and Discussion

### 3.1. Microscopic Observations of the Original Consortia

**Figure 2:**
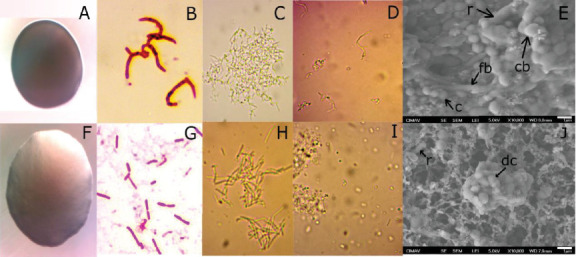
Observation of the consortia: (A–E) for C55 and (F–J) for C33. The first column (A, F) shows the colonies grown on LB solid media: C55 and C33 colony sizes varied between 1 and 1.5 mm. The next three columns reproduce cell observations by OM (magnification 1000×): gram stain in the second column (B, G), liquid culture without Cr(VI) (C, H), and liquid culture with 300 mg·L^−1^ Cr(VI) (D, I). The last column (E, J) shows SEM images of LB liquid culture without Cr(VI) showing details of cell morphologies: (r) rod-shaped, (c) coccus-shaped, (cb) coccobacillus, (dc) diplococci, and (fb) elongated bacilli forming filaments (35,000 × magnification and bar =1 *μ*m).

OM revealed typical filamentous growth of C55 consortium cells in liquid medium (Figure 2B,C), which was quite different from that observed for the C33 consortium, which was formed by rod-shaped cells (Figure 2G,H). SEM images revealed different cellular morphologies: rod-shaped (r), elongated bacilli (fb), coccobacilli (cb), and diplococci-like (dc) cells (Figure 2E,J). By increasing the chromate concentration (to 300 mg·L^−1^), the development of diplococcal cells and spore structures was favored on both cultures (Figure 2D,I). The C55 consortium showed resistance up to 800 mg·L^−1^ Cr(VI), but the ability to decrease the amount of Cr(VI) in the medium was not observed above 400 mg·L^−1^. The C33 consortium showed resistance up to 400 mg·L^−1^ and the capacity to decrease Cr(VI) of the medium up to 200 mg·L^−1^.

### 3.2. Biodiversity of Consortia

**Figure 3:**
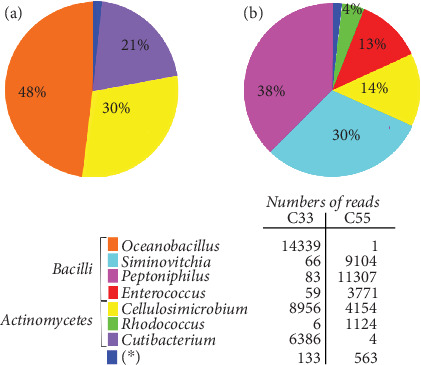
Bacterial diversity based on V4–V5 region of 16S rRNA. Pie diagrams showing the relative abundance of OTUs at the genus level: (a) C33 consortium and (b) C55 consortium samples. ⁣^∗^OTUs with less than 1% of relative abundance.

High-throughput sequencing of both consortium samples revealed 60,056 reads with a length of approximately 400 bp. Biodiversity analysis was carried out by comparing our sequences with those in the Silva database (version 138.1). Eight operational taxonomic units (OTUs) were detected in the C33 consortium, and 14 were detected in the C55 consortium; these OTUs were distributed mostly in two phyla (Actinomycetota and Bacillota). The majority populations (abundances above 10%) for C33 and C55 were represented by 3 (98.9% of the abundance) and 4 (94.4%) genera, respectively, as shown in Figure 3. These were affiliated with *Cellulosimicrobium* (major in both), *Oceanobacillus* and *Cutibacterium* (major only in C33), and *Peptoniphilus*, *Siminovitchia*, and *Enterococcus* (major only in C55), while *Rhodococcus* presented significant abundance (above 1%) only in the C55 consortium.

Previous bacterial diversity analyses of the original sample site (a residue from the chromite processing industry) sampled in 2008 and 2014 revealed a relatively high abundance of the Bacillota phylum (Bacilli, Negativicutes, and Clostridia classes), followed by Pseudomonadota (Gammaproteobacteria, Betaproteobacteria, and Alphaproteobacteria classes), and Bacteroidota (Bacteroidia class), while the Actinomycetota phylum showed a low abundance (between 2.8% and 4.1%) [15, 23]. Thus, the composition of the original samples was distinct from that observed for the consortia studied here. Most likely, the composition of the culture medium used in the enrichment before obtaining these consortia influenced the results, favoring the selection of spore-producing communities. For example, populations of the Bacillota phylum were preferentially selected in comparison with populations of the Pseudomonadota phylum. On the other hand, the Actinomycetota phylum, even with a low representation in the original sample, showed a high representation in both consortia. Notably, one of the criteria used in selecting these consortia was their resistance to chromate ions, which likely influenced the selection of these populations. In fact, both the Bacillota and Actinomycetota phyla have been widely reported as potential bioremediation agents for the environmental mitigation of Cr(VI) [31].

### 3.3. Isolated Bacteria

**Figure 4:**
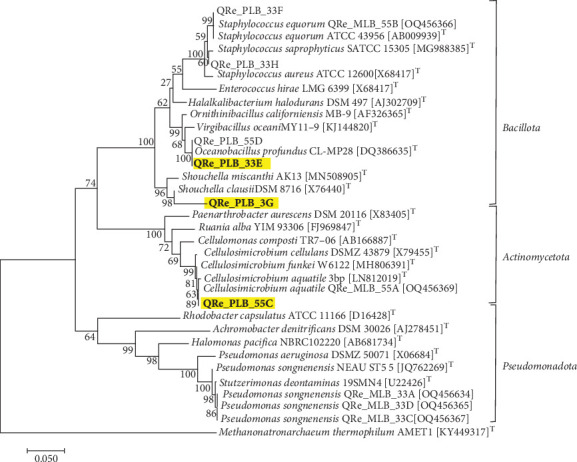
Phylogenetic tree based on 16S rRNA encoding gene, showing the position of our isolated strains (in bold letters) within the radius of members of respective groups: Bacillota and Actinomycetota. The accession numbers of all type strains are indicated in brackets. The yellow squares highlight the strains selected for the Cr(VI) assays. The tree is constructed using MEGA11 software. Branch lengths, drawn to scale, are measured as the number of substitutions per site, which were obtained automatically applying Neighbor-Joining and BioNJ algorithms to a matrix of pairwise distances estimated using the Tamura-Nei model. Reliability of the inferred tree is estimated by a bootstrap test with 1000 resamplings. This analysis involved 33 nucleotide sequences of 1381 bp.

Although the main interest of this work is to study the cooperation among different populations (specifically from the two consortia C33 and C55), in the search for a combination with an amplified capacity for diminishing Cr(VI), we also submitted our consortia to an isolation study. Four cultivable strains from the C33 consortium and two from the C55 consortium were obtained. Sanger analysis revealed that these six strains are distributed in the phyla Actinomycetota and Bacillota (Figure 4), as expected.

The isolates QRePLB33E and QRePLB55D showed the highest similarity to *Oceanobacillus profundus* (100.0% and 97.7%, respectively). *O. profundus* is a facultative alkaliphilic bacterium isolated from a marine sediment core sample collected at a depth of 2247 m in the Ulleung Basin of the East Sea, Korea [32]. Recently, Mwandira et al. [33] showed that a strain of this species (*O. profundus* KBZ 3-2) can remove Pb and Zn from water samples through a biosorption mechanism. As will be presented below, this is the first time that a representative of this species has been shown to be able to decrease Cr(VI) in the medium. On the other hand, Zeng et al. [4] demonstrated that another strain of this genus, *Oceanobacillus oncorhynchi* W4, is capable of removing Cr(VI) from solution in 3 days (74% at an initial Cr(VI) concentration of 200 mg·L^−1^). At the end of the experiment, chromium adhered to the cell surface, forming organic complexes and Cr(OH)_3_. From our two strains of this genus, we selected QRePLB33E to verify its resistance to and ability to reduce Cr(VI) in liquid medium.

The isolate **QRePLB33G** showed the highest similarity (94.2%) to *Alkalihalobacillus clausii* [34], which was recently reclassified as *Shouchella clausii* (Clade V) by Joshi et al. [35]. The *Alkalihalobacillus* genus is composed of extremophilic bacteria (alkaliphilic and halotolerant/halophilic). Several species of this genus are of great interest due to their ability to produce enzymes that are potentially applicable to industry [34]. Currently, two strains of this species are identified to be resistant to several metals: *Bacillus clausii* S6-04, which is resistant to Co, Se, Hg, Mn, As, Cu, Mg, Cd, and Pb [36], and *A. clausii* CRA1, which is resistant to Cr, Cd, As, Pb, Ni, Hg, Cu, Zn, and Fe [28]. *A. clausii* CRA1 was isolated from tannery effluent and showed the ability to decrease chromate ions by 72% in 6 days from an initial Cr(VI) concentration of 50 mg·L^−1^ [28].

The isolate **QRePLB55C** showed close similarity to two species of the genus *Cellulosimicrobium* (*Cellulosimicrobium funkei* and *Cellulosimicrobium aquatile*, 98.04% and 97.96%, respectively). Previously, we isolated two other strains (*C. funkei* strain **QRePRA55** and *C. aquatile* strain **QReMLB55A**) from the same site [15]. Both isolates showed resistance to 400 mg·L^−1^ and were also able to decrease the ion concentration in liquid medium [17, 37]. Currently, the Cr(VI) resistance observed in microorganisms of the genus *Cellulosimicrobium* has attracted much attention. For example, Bathi et al. [38] determined a sequential order for toxic metal remediation capacity carried out by a *Cellulosimicrobium* sp. that was iron > lead > zinc > copper > nickel > cadmium. Additionally, some strains of this genus isolated from tannery wastewater have been shown to be resistant to chromium, able to promote plant growth [39], or capable of reducing Cr(VI) to Cr(III) [40]. The overexpression of metal transporter NRAMP genes and stress marker genes in plants was observed when both *C. funkei* AR6 and Cr(VI) were inoculated in soil [3, 41, 42]. In 2001, the genus *Cellulomonas* was reclassified to *Cellulosimicrobium*, encompassing several microorganisms of the Actinomycetota phylum with common phylogenetic and metabolic characteristics [43]. In this genus, they include chemo-organotrophic bacteria with cellulolytic activity, which are composed of gram-positive bacteria with nonmotile cells that do not form endospores. In fresh cultures, mycelial-like growth is observed with elongated, curved, sometimes V-shaped cells. After exhaustion of the medium, the rods transform into shorter rods or even spherical cells [43]. All these characteristics were observed in the C55 consortium culture and in the cultures of the isolates **QReMLB55A**, **QRePLB55C**, and **QRePRA55**.

The isolates **QRePLB33F** and **QRePLB33H** showed similarity to *Staphylococcus equorum* and *Staphylococcus saprophyticus* (100.0% and 99.8%, respectively). Although no staphylococcal pathogenicity or virulence factors were attributed to the *equorum* species, a comparative genomic study revealed specific determinants linked to phenotypic traits of virulence and salt tolerance [44], encouraging us to discard these determinants from this study.

Microbial prospecting of metal-polluted environments has yielded diverse bacterial strains capable of metal biotransformation (e.g., [45]). While significant progress has been made in understanding these mechanisms [46], many aspects remain unclear. In the case of chromate, several bacterial genera have been reported for their ability to reduce the toxic Cr(VI) to the less toxic Cr(III) (for a review, see [47]). However, different species within the same genus may not necessarily exhibit this ability, and even strains within the same species can vary in their capacity to perform this reduction. This variability is likely due to diverse mechanisms employed by microorganisms to interact with and process pollutants.

Cr(VI) reduction can occur through enzymatic pathways triggered by genes like chromate resistance determinants, which are induced in the presence of chromate [48, 49]. This process can take place under both aerobic and anaerobic conditions. Aerobic reduction is often associated with soluble chromate reductases [50], while anaerobic reduction involves the use of Cr(VI) as an electron acceptor in the electron transport chain [51]. Additionally, Cr(VI) can be reduced indirectly through nonspecific reactions with redox-active organic compounds like amino acids, nucleotides, and sugars [52, 53].

### 3.4. Synergy and Chromate Ion Diminution/Depletion

#### 3.4.1. Growth Rates of the Original and Mixed Consortia

**Figure 5:**
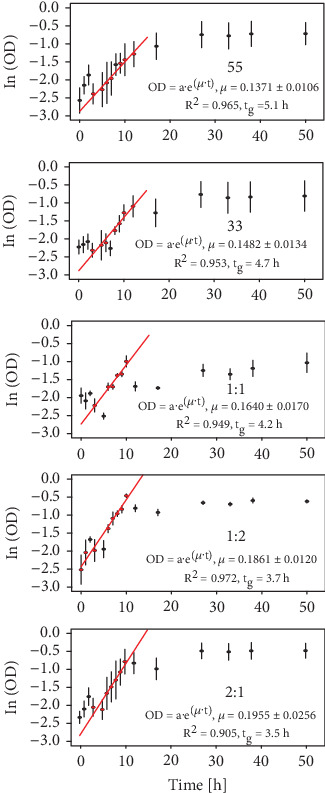
Bacterial growth rates (*μ*) in the exponential phase (in LB liquid media) using different proportions of the C55 and C33 consortia as *inocula*. All the systems began with 10^7^ cel·mL^−1^. All experiments were performed in triplicate, and OD was measured at 600 nm.

The possible bacterial synergism between the populations of the two consortia (C55 and C33) was first evaluated by measuring *μ* and *t*_g_ in LB medium and in the absence of the xenobiotic, for them separately and for their mixtures C11, C21, and C12 (Figure 5**)**. As shown in this figure, the three mixtures had higher *μ* values than did the individual original consortia, indicating a lower *t*_g_, a desirable condition for their use in bioprocesses.

#### 3.4.2. Chromate Depletion by the Original and Mixed Consortia

**Figure 6:**
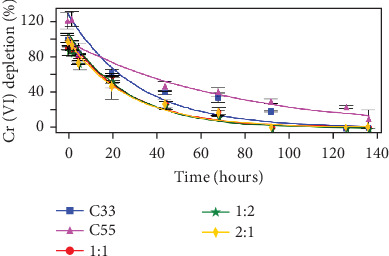
Ability of the C55 and C33 consortia and different mixtures to decrease the amount of chromate ions at 50 mg·L^−1^ Cr(VI). Proportions (C55:C33): red dots, 1:1; green stars, 1:2; and yellow diamonds, 2:1. All systems started with 10^7^ cel·mL^−1^ as *inoculum*, the controls consisted of LB medium without *inoculum,* and three replicates were prepared for all the studied conditions. The data shown were normalized to the respective controls.

**Figure 7:**
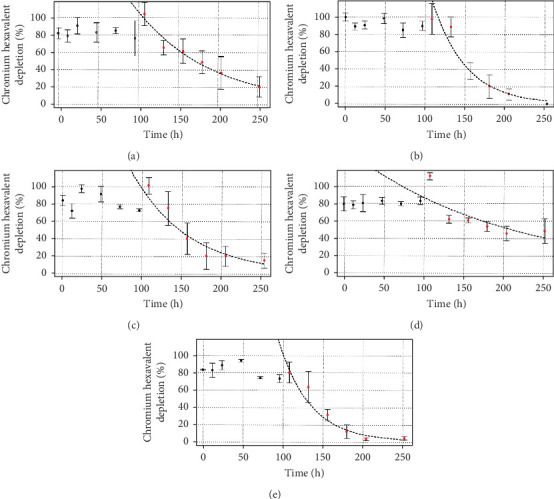
Ability to decrease chromate ion by C55 and C33 consortia and different mixtures at 100 mg·L^−1^ Cr(VI). (a) C33 consortium, (b) C55 consortium, (c) proportion 1:1 (C55:C33), (d) proportion 1:2 (C55:C33), and (e) proportion 2:1 (C55:C33). All systems started with 10^7^ cel·mL^−1^ as *inoculum*, the controls consisted of LB medium without *inoculum*, and three replicates were prepared for all the studied conditions. The data shown have been normalized by the control. The Cr(VI) decreasing rate was calculated by applying a logarithmic decay regression to the red data points only.

**Figure 8:**
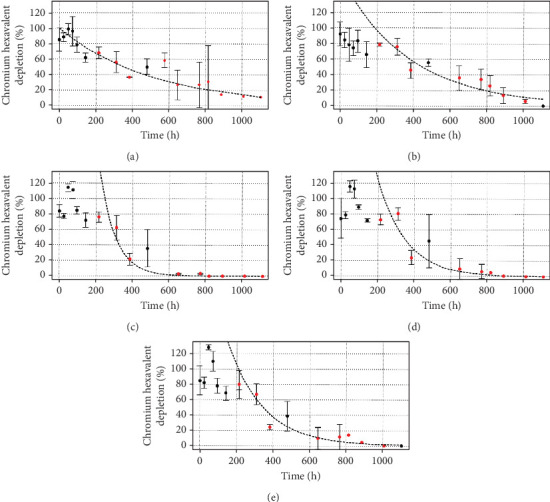
Ability to decrease chromate ion by C55 and C33 consortia and different mixtures at 200 mg·L^−1^ Cr(VI). (a) C33 consortium, (b) C55 consortium, (c) proportion 1:1 (C55:C33), (d) proportion 1:2 (C55:C33), and (e) proportion 2:1 (C55:C33). All systems started with 10^7^ cel·mL^−1^ as *inoculum*, the controls consisted of LB medium without *inoculum*, and three replicates were prepared for all the studied conditions. The data shown have been normalized by the control. The Cr(VI) decreasing rate was calculated by applying a logarithmic decay regression to the red data points only.

For Cr(VI) depletion experiments, the same five consortia (originals and mixtures) were evaluated at 50, 100, and 200 mg·L^−1^ Cr(VI), together with their respective controls (culture media without microorganisms but with the xenobiotic— percentage decrease with respect to the control values is shown in the first three figures). As expected, lower reduction rates were observed for systems with higher chromium contents (Table 1). All the mixtures presented better results than did the original consortia for 50 mg·L^−1^ Cr(VI) (Figure 6), while no apparent difference between them was observed, presenting a calculated kinetic constant *k* varying between 0.0416 and 0.0427, resulting in a *t*_1/2_ for Cr(VI) depletion from the media of approximately 16 h. This was not the case for 100 mg·L^−1^ Cr(VI) (Figure 7), for which the C55 consortium and the C21 mixture showed the best *k* values, with a *t*_1/2_ for Cr(VI) depletion of approximately 20 h, followed by the C11 mixture (*t*_1/2_ of approximately 30 h). Finally, for 200 mg·L^−1^ Cr(VI) (Figure 8), the highest rate of chromate ion depletion in the medium was observed for the C11 mixture (*t*_1/2_ of approximately 100 h). Thus, we conclude that the mixture consortia performed better at depleting chromate ions than did the original consortia, with C11 presenting the best results among the three tested concentrations.

Comparing the ion complete removal times, considering the most efficient conditions for Cr(VI) depletion, we observed a clear correlation between initial Cr(VI) concentration and treatment duration: 50 ppm (approximately 92 h for the three mixture consortia), 100 ppm (approximately 204 h for C21), and 200 ppm (approximately 600–800 h for C11). Doubling the Cr(VI) concentration roughly doubled or tripled the depletion time, as evident in the half-life calculations (for example, for C11). Furthermore, increasing Cr(VI) concentrations required longer adaptation periods (Figures 6, 7, and 8). No adaptation phase was necessary at 50 mg·L^−1^, while approximately 4.5 and 9 days were needed for 100 and 200 mg·L^−1^, respectively. These findings align with previous studies that reported prolonged lag phases, decreased growth rate of the bacterial cells, accompanied by morphological changes and increased stress responses at higher Cr(VI) concentrations [54–56]. We also observed noticeable morphological changes in cells (Figure 2D,I) as Cr(VI) concentrations increased, indicating cellular stress and toxicity [54, 57]. For instance, the C55 consortium exhibited elongated cells forming aggregates, similar to *Enterobacter cloacae* strain B2-DH when exposed to higher Cr(VI) concentrations [57]. This morphological adaptation is likely a strategy for metal accumulation and stress mitigation [58].

### 3.5. Chromate Depletion by Isolated Strains

**Figure 9:**
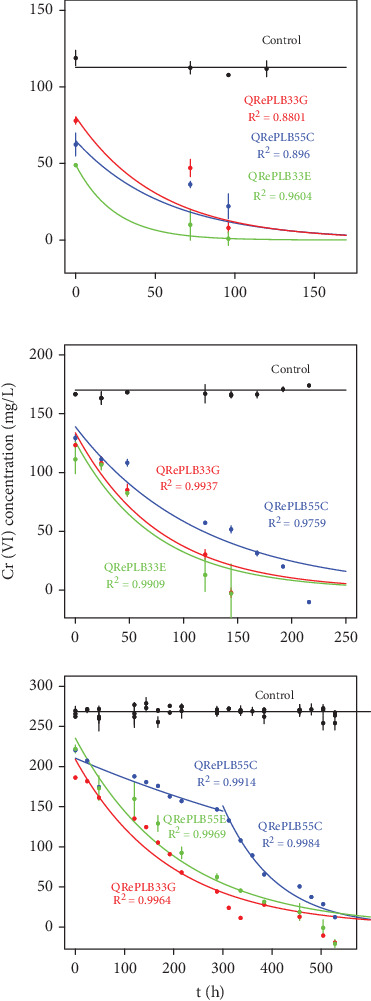
Chromate depletion experiment for the three selected isolates: QRePLB33E (green), QRePLB33G (red) and QRePLB55C (blue), at three different Cr(VI) initial concentrations: 113, 170, and 268 mg·L^−1^.

We also analyzed the ability of the three selected isolates to completely eliminate Cr(VI) from liquid medium. The results of this experiment are presented in the panels of Figure 9. We found that **QRePLB33E** was able to deplete 100% of Cr(VI) at 6, 8, and 26 days at approximately 100, 200, and 300 mg·L^−1^, respectively. For the **QRePLB33G** isolate, we observed a 100% decrease in Cr(VI) at 5, 8, and 16 days, again at approximately 100, 200, and 300 mg·L^−1^. Finally, the strain **QRePLB55C** could eliminate chromate ions at 5, 15, and 25 days. As expected (see the review [58]), the greater the Cr(VI) concentration was, the more time was needed for each strain to eliminate the xenobiotic.

The respective decay rates and half-lives obtained for the three strains under the three Cr(VI) conditions are shown in Table 2.

One can see that **QRePLB33E** showed a better performance at 113 and 170 mg·L^−1^ of Cr(VI) (in the latter, very similar to **QRePLB33G**), while for 268 mg·L^−1^, **QRePLB55C** revealed a complex pattern with two decaying phases.

These results were better than those observed for *A. clausii* CRA1 at a Cr(VI) concentration of 50 mg·L^−1^ [28]. Such strain was able to diminish 72% of the Cr(VI) in 144 h while our mixed consortia removed 100% of this ion from the liquid media in 92 h, and also, our isolated strain similar to this specie (**QRePLB33G**) was able to deplete Cr(VI) in approximately the same 144 h. Several bacterial strains can eliminate Cr(VI) from media (for a review, see [59]). Among these, strains of the genus *Cellulosimicrobium* exhibit resistance to up to 800 mg·L^−1^ Cr(VI) and are able to remove chromium from the medium at concentrations as high as 300 mg·L^−1^ [60]. Our strain of this genus (**QRePLB55C**) exhibited a depletion time of approximately 500 h at a concentration of 268 mg·L^−1^. The genus *Oceanobacillus* [4] also deserves mention here due to its presence in our consortia (**QRePLB33E**), with excellent performance for the three tested Cr(VI) concentrations.

### 3.6. Biofilm Formation Capacity of the Consortia

**Figure 10:**
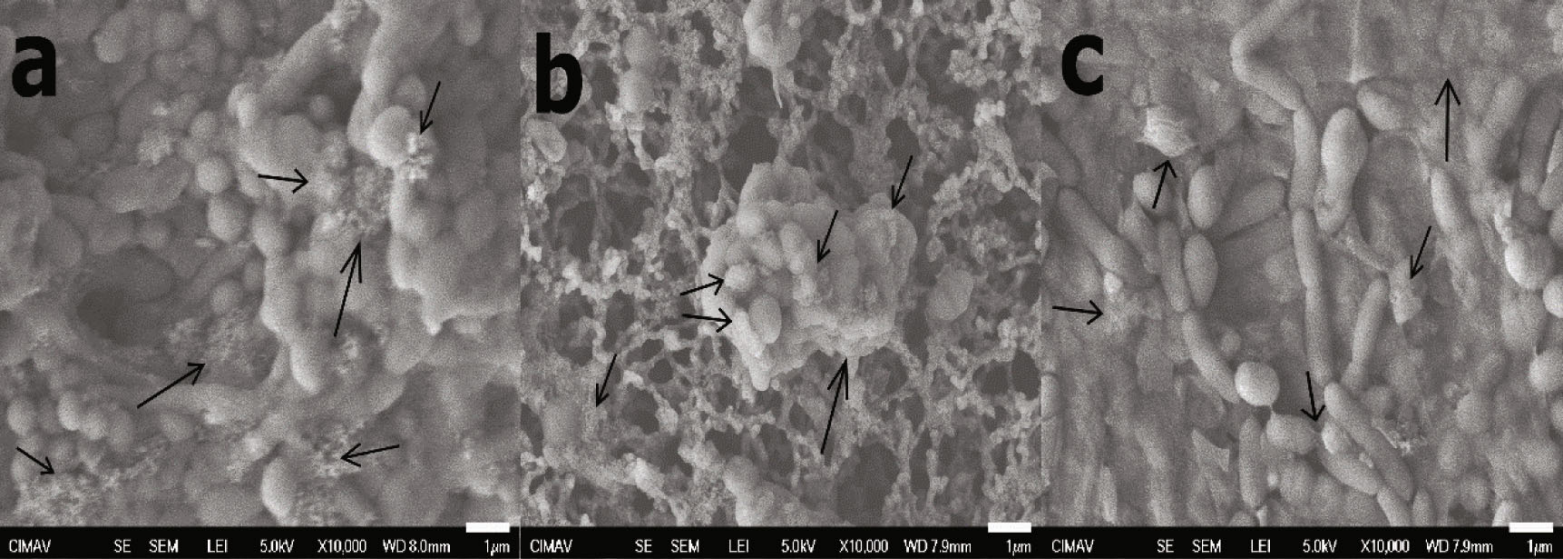
SEM images of biofilm development in liquid culture media: (a) C55, (b) C33, and (c) C21 consortia. Polymeric substance (EPS) over the cells is pointed by the arrows. Greater development of biofilms and cells can be observed in the mixture of the consortia (c) (35,000 × magnification and bar =1 *μ*m).

The ability to form biofilms was first checked by visualizing the adherence of the cells using safranin staining (on a microtiter plate of polystyrene). From the original consortia, only C33 showed strong staining, suggesting the ability to grow producing biofilm, while for C55, the color was very weak or absent. These observations were confirmed by SEM analysis of these cultures (Figure 10). Apparently, the C55 consortium can produce some polymeric substances (EPSs) but in much smaller amounts than the C33 consortium can (Figure 10a,b). In turn, the mixture of C33 with C55 consortia (C21) showed an increase in the production of polymeric substances (Figure 10c). A greater number of cells was also observed in this mixture than in the individual consortia.

Halophilic microorganisms are known for their ability to form biofilms and to accumulate EPS under stress conditions [61]. Both of our consortia are haloalkaliphilic (see Section 2.1). In addition, three of the detected genera conforming C33 consortium (*Oceanobacillus*, *Cellulosimicrobium*, and *Cutibacterium*) have demonstrated the ability to produce biofilms [14, 62]. Among these, populations of the *Oceanobacillus* genus were studied as potential sources of new biomolecules. For example, the bioflocculant MBF-HG6 was obtained from *Oceanobacillus polygoni* and was subsequently characterized as containing 81.53% polysaccharides and 9.98% proteins [14]. For C55, on the other hand, we detected populations similar to those of the genus *Siminovitchia* (with 30% relative abundance). Some microorganisms belonging to this genus, *such as Bacillus sediminis*, have been isolated from biofilms [63]. *Siminovitchia* is a new genus that was proposed for the reclassification of *Bacilli* species by Gupta et al. [64]. In this genus, five older *Bacillus* species (*Bacillus acidinfaciens*, *Bacillus composti*, *Bacillus farraginis*, *B. sediminis*, and *Bacillus thermophilus*) are grouped. The C55 consortium also contained potentially pathogenic bacterial genera, such as *Enterococcus* (with 13% total relative abundance) and *Peptoniphilus* (38%). Microorganisms of these genera can grow biofilms. For example, *Enterococcus faecalis and Enterococcus faecium* can colonize a wide variety of medical devices to form biofilms and are associated with various human diseases [65].

The ability to produce EPS by the studied consortia is a promising physiological characteristic for the development of Cr(VI) mitigation bioprocesses. SEM analysis with EDX detector of bacteria exposed to Cr(VI) revealed metal association with EPS [66]. Several studies have shown that the chemical composition of EPS can influence metal accumulation. For instance, EPS rich in uronic acid and acidic sugars tends to accumulate higher amounts of Cr(VI) [67, 68]. Furthermore, the presence of metal can stimulate EPS production.

Another example of this phenomenon was observed by Zhou et al. [69], who investigated EPS characteristics and variations in response to Cr(VI) at concentrations ranging from 20 to 125 mg·L^−1^, using a fixed-bed reactor (0.8 L). They observed that as Cr(VI) concentration increased from 0 to 60 mg·L^−1^, the EPS content increased. However, at 80 mg·L^−1^, there was a decline in total EPS content. Interestingly, the introduction of a recovery stage in their experiment led to a rapid renewal of microbial activity and biofilm formation. They also noted a reduction in protein and polysaccharide content in EPS after Cr(VI) addition, which could impact bacterial surface characteristics and cell aggregation [70]. They hypothesized that continuous exposure to Cr(VI) and its accumulation could stimulate microbes to secrete more proteins by adjusting their metabolic activities [1, 50]. Since enzymes are predominantly composed of proteins, the proteins in EPS may contain reductases capable of reducing Cr(VI) to Cr(III). Although we did not study the biofilm–Cr(VI) interaction in depth in this work, the ability of microorganisms to produce biofilms and transform Cr(VI) under axenic culture conditions, particularly in the presence of high chromium concentrations, provides useful insights into this topic. This interaction remains an area for further investigation.

## 4. Conclusions

The two original consortia, C33 and C55, showed phylogenetic compositions, with 9 and 11 OTUs, respectively, distributed in the Actinomycetota (mostly *Cellulosimicrobium*, *Cutibacterium*, and *Rhodococcus* genera) and Bacillota (mostly *Oceanobacillus*, *Peptoniphilus*, *Siminovitchia*, and *Enterococcus* genera) phyla. As expected, the isolated strains were also affiliated with *Cellulosimicrobium*, *Oceanobacillus*, and *Staphylococcus* (minority in the metagenomic profile), together with one similar to the *Alkalihalobacillus/Shouchella* genus (not detected in the original consortia profile).

Confirming our original hypothesis, the mixture consortia outperformed the original consortia in decreasing Cr(VI) from the medium. The composition of the bacterial community is responsible for the differing responses to stress conditions. Thus, mixtures of consortia with different population compositions, capabilities, and resistances to xenobiotics may, at higher concentrations, collaborate with each other, allowing for greater xenobiotic depletion and resulting in a synergistic effect. Additionally, the efficiencies of the bacterial consortia in reducing Cr(VI) in the medium were similar to those of the strains from the same consortia.

The synergistic effects observed in the mixtures are attributed to the diversity in population composition, functional capabilities, and resilience to stress conditions. By combining consortia with distinct traits, the mixture allowed for improved xenobiotic depletion, likely facilitated by cooperative interactions among the microbial populations.

Concerning biofilm formation, we also verified that the original C33 consortium is more capable of producing EPS than is C55. This biofilm may enhance the Cr(VI) depletion rate by providing a structural advantage and potentially contributing to higher tolerance to elevated Cr(VI) concentrations. Future work will explore strategies to optimize biofilm production, such as varying carbon sources, to improve the consortia's efficiency at higher contaminant concentrations. The next stages of research will also focus on assessing these consortia under scaled-up conditions, evaluating their long-term stability, and conducting detailed Cr(VI) speciation studies to confirm chromium immobilization pathways. A preliminary study carried out in this direction suggested that Cr(VI) is being absorbed/adsorbed by the biomass since total chromium was not detected at the end of the experiment (in the liquid medium). These steps are crucial for translating the current laboratory findings into scalable, sustainable bioprocesses for Cr(VI) mitigation in contaminated environments. From a practical perspective, the findings of this study provide foundational insights into the microbial dynamics and mechanisms underlying Cr(VI) depletion.

## Figures and Tables

**Table 1:** The kinetic constant *k* for the first-order reaction was calculated by applying a logarithmic decay regression to the data in Figures 6, 7, and 8. The half-life (*t*_1/2_) is measured in hours.

**Consortia and mixtures (C55:C33)**	**Systems at different Cr(VI) concentrations**								
	**50 mg·L** ^**−1**^			**100 mg·L** ^**−1**^			**200 mg·L** ^**−1**^		
	**k**	**R** ^2^	**t**_1/2_	**k**	**R** ^2^	**t**_1/2_	**k**	**R** ^2^	**t**_1/2_
C55	0.0138	0.881	50	**0.0320**	0.950	**21**	0.0038	0.752	183
C33	0.0297	0.843	23	0.0107	0.978	65	0.0020	0.974	346
C11	**0.0416**	0.870	**16**	**0.0228**	0.974	**31**	**0.0071**	0.964	**98**
C21	**0.0418**	0.880	**16**	**0.0352**	0.932	**20**	0.0051	0.958	136
C12	**0.0427**	0.889	**16**	0.0050	0.663	139	0.0055	0.916	126

*Note:* The most expressive decay rates are highlighted in bold.

**Table 2:** The kinetic constant *k* for the first-order reaction was calculated by applying a logarithmic decay regression to the data in Figure 9. The half-life (*t*_1/2_) is measured in hours.

**Isolates**	**Systems at different Cr(VI) concentrations**					
	**113 mg·L** ^**−1**^		**170 mg·L** ^**−1**^		**268 mg·L** ^**−1**^	
	**k**	**t**_1/2_	**k**	**t**_1/2_	**k**	**t**_1/2_
QRePLB33E	0.0404	17	0.0136	51	0.0049	142
QRePLB33G	0.0192	36	0.0128	54	0.0053	131
QRePLB55C	0.0176	39	0.0087	80	0.0092	(300 + 75)^∗^

⁣^∗^Low, almost linear decay before 300 h, exponential decay after that.

## Data Availability

The datasets generated and analyzed during the current study were deposited in the Sequence Read Archive (SRA) database of the National Center for Biotechnology Information (NCBI) which assigned accession numbers were associated with the project PRJNA1121098, while the 16S rDNA sequences (determined by Sanger approach) were deposited at GenBank database (assigned Accession Numbers PP747663–PP747668).
